# Supplementary material for: Magnetique: an interactive web application to explore transcriptome signatures of heart failure
Source: J Transl Med. 2022 Nov 7;20:513. doi: 10.1186/s12967-022-03694-z (PMC9641957; doi:10.1186/s12967-022-03694-z)
Supplement: Supplementary file 1 — Supplementary Material 1 [file 12967_2022_3694_MOESM1_ESM.pdf]

Magnetique: A web application for interactive Transcriptome Exploration in Heart Failure

Thiago Britto-Borges, Annekathrin Ludt, Etienne Boileau, Enio Gjerga, Federico Marini, Christoph Dieterich

	Overall, N = 354[1]	DCM, N = 165[1]	HCM, N = 27[1]	NFD, N = 162[1]	p-value[2]
**Race**					<0.001
AA	121 (34%)	76 (46%)	1 (3.7%)	44 (27%)	
C	233 (66%)	89 (54%)	26 (96%)	118 (73%)	
**Age**	56 (48, 62)	54 (47, 59)	51 (43, 57)	57 (51, 65)	<0.001
**Sex**					0.021
F	164 (46%)	65 (39%)	11 (41%)	88 (54%)	
M	190 (54%)	100 (61%)	16 (59%)	74 (46%)	

Weight	79 (66, 91)	76 (67, 90)	72 (60, 83)	82 (68, 99)	0.004
Height	170 (163, 178)	170 (163, 180)	170 (160, 180)	168 (163, 175)	0.046
Unknown	1	0	0	1	
HW	448 (372, 558)	499 (419, 603)	448 (366, 559)	414 (345, 495)	<0.001
Unknown	7	0	0	7	
LVMass	247 (199, 322)	320 (262, 401)	318 (276, 340)	214 (179, 278)	<0.001
Unknown	202	119	15	68	
AFib	104 (30%)	67 (41%)	20 (74%)	17 (11%)	<0.001
Unknown	4	3	0	1	
VTVF	85 (24%)	70 (43%)	14 (52%)	1 (0.6%)	<0.001
Unknown	2	1	0	1	
Diabetes	76 (22%)	39 (24%)	0 (0%)	37 (23%)	0.018

Unknown	2	0	0	2	
Hypertension	85 (24%)	70 (43%)	14 (52%)	1 (0.6%)	<0.001
Unknown	2	1	0	1	
LVEF	0.20 (0.15, 0.52)	0.15 (0.10, 0.20)	0.25 (0.16, 0.42)	0.55 (0.50, 0.65)	<0.001
Unknown	78	5	1	72	
TIN	70 (61, 73)	69 (57, 73)	71 (63, 74)	71 (65, 74)	0.019
RIN	8.50 (8.10, 8.90)	8.30 (7.80, 8.80)	8.70 (8.45, 8.85)	8.70 (8.20, 9.10)	<0.001
Unknown	6	1	0	5	
DuplicationRate	0.42 (0.38, 0.52)	0.42 (0.38, 0.52)	0.39 (0.35, 0.53)	0.43 (0.38, 0.51)	0.6
**SV1**	0.02 (-0.03, 0.04)	0.02 (-0.05, 0.04)	0.04 (-0.02, 0.04)	0.02 (-0.01, 0.04)	0.4
**SV2**	-0.02 (-0.04, 0.02)	-0.01 (-0.03, 0.02)	-0.01 (-0.04, 0.02)	-0.02 (-0.04, 0.01)	0.4
[1] Median (IQR); n (%)					

[2] Kruskal-Wallis rank sum test; Pearson's Chi-squared test
Bold highlight features were used in the statistical model.
Acronyms: HW: Heart weight; TIN: Transcript Integrity Number: RIN: RNA integrity number; LVMass: Left ventricular mass; AFib: Atrial fibrillation events; VTVF: Ventricular tachycardia/ventricular fibrillation; LVEF: Left ventricular ejection fraction;

Table S01: Patient characteristics and sample metadata.

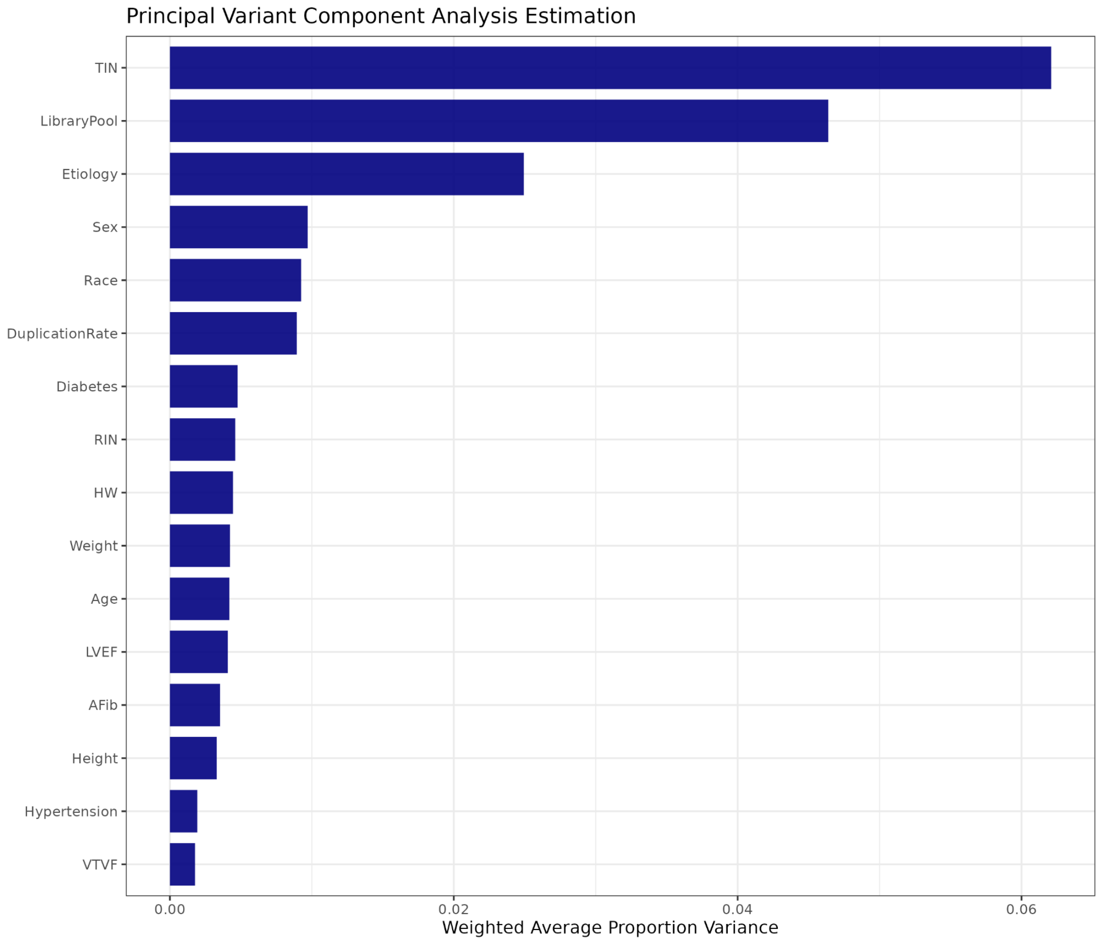

Figure S01. Component analysis for covariates in the MAGnet RNA-Seq dataset. The residuals (based on the full model fit) explain most of the proportion of variance (0.80) in the data. The contribution of the known model covariates to explaining data variance is shown in this figure. For example, Transcript Integrity Number (TIN) and LibraryPool (batch for library sequence) explain more variation in the data than sex.

**Software**	**Version**	**Repository**
Flexbar	3.5.0	https://github.com/seqan/flexbar
Bowtie2	2.3.5.1	https://github.com/BenLangmead/bowtie2
STAR	2.6.0c	https://github.com/alexdobin/STAR/
Picard tools	2.22.1	https://github.com/broadinstitute/picard
StringTie2	2.1.3b	https://github.com/gpertea/stringtie

sva	3.38.0	https://github.com/jtleek/sva
pvca	1.30.0	https://github.com/dleelab/pvca
DESeq2	1.30.1	https://github.com/mikelove/DESeq2
GeneTonic	1.7.3	https://github.com/federicomarini/GeneTonic
topGO	2.42.0	https://bioconductor.org/packages/release/bioc/html/topGO.html
Shiny	1.7.1	https://github.com/rstudio/shiny
tidyverse	1.3.1	https://github.com/tidyverse/tidyverse
dplyr	1.0.7	https://github.com/tidyverse/dplyr
ggplot2	3.3.5	https://github.com/tidyverse/ggplot2
GenomicRanges	1.46.0	https://github.com/Bioconductor/GenomicRanges
rtracklayer	1.54.0	https://github.com/lawremi/rtracklayer
DRIMSeq	1.18.0	https://github.com/gosianow/DRIMSeq
DoRothEA	1.7.0	https://github.com/saezlab/dorothea
edgeR	3.32.1	https://bioconductor.org/packages/release/bioc/html/edgeR.html
BiRewire	3.26.5	http://bioconductor.org/packages/release/bioc/html/BiRewire.html
VIPER	1.28.0	https://www.bioconductor.org/packages/release/bioc/html/viper.html
OmnipathR	3.3.20	https://bioconductor.org/packages/release/bioc/html/OmnipathR.html
CARNIVAL	2.4.0	https://github.com/saezlab/CARNIVAL
globaltest	5.44.0	https://www.bioconductor.org/packages/release/bioc/html/globaltest.html

Table S02. Software used in this work. The table lists the software, software version, and repository link used on the manuscript.

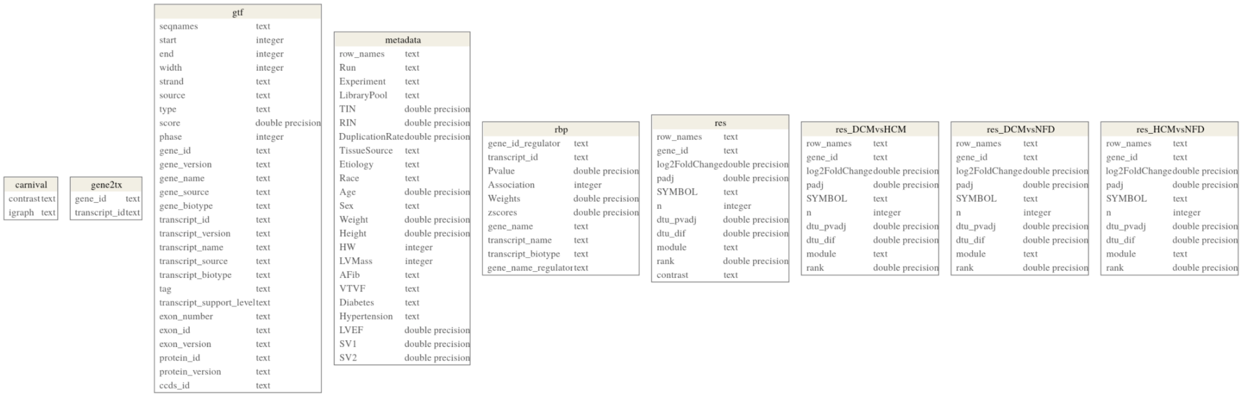

Figure S02. Magnetique PostgreSQL schema. The first column in each table represents the column name, and the second column represents the data type. The matrices of features for each patient are represented by three additional tables, named counts (gene counts), dtu fit proportions (fitted transcript proportions), and vst (variance stabilizing transformed gene counts), which were not included in the scheme.

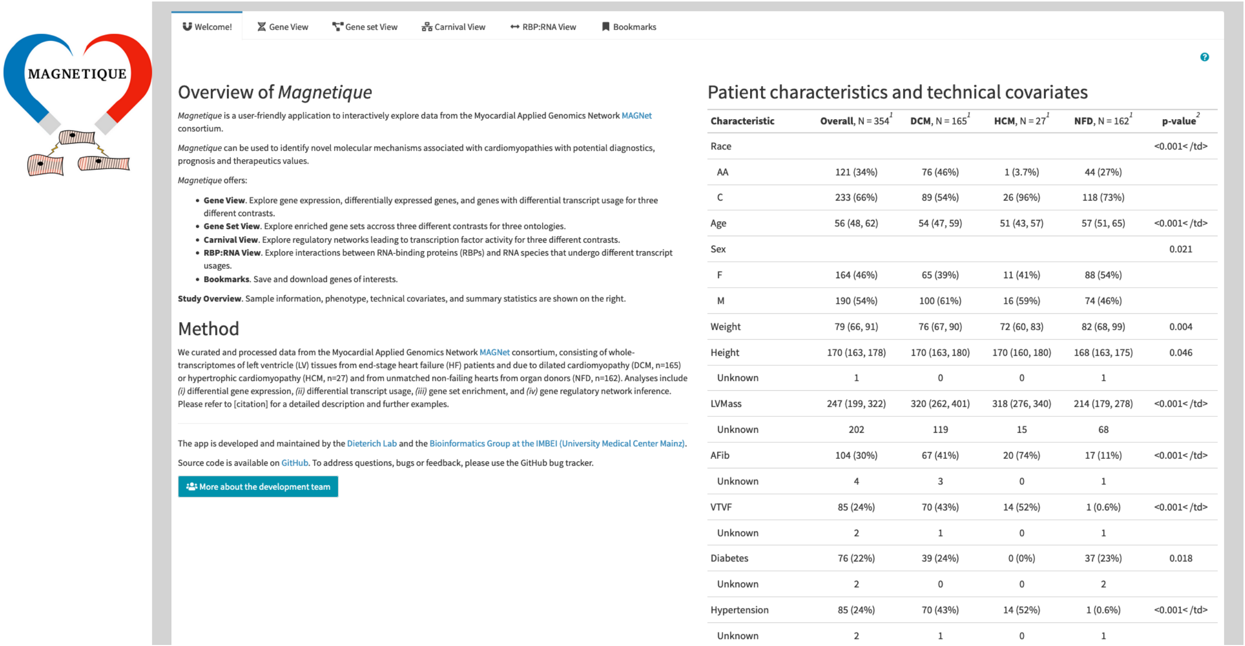

Figure S03. Partial screenshot of the landing page for the Magnetique application. The image shows the landing page for the Magnetique application. The different views can be accessed by the multiple tabs at the top. The Welcome tab is followed by the Gene, Gene set, and Carnival views. The help pages are specific to each tab and can be accessed by the interrogation mark at the upper right section on the page (not shown).

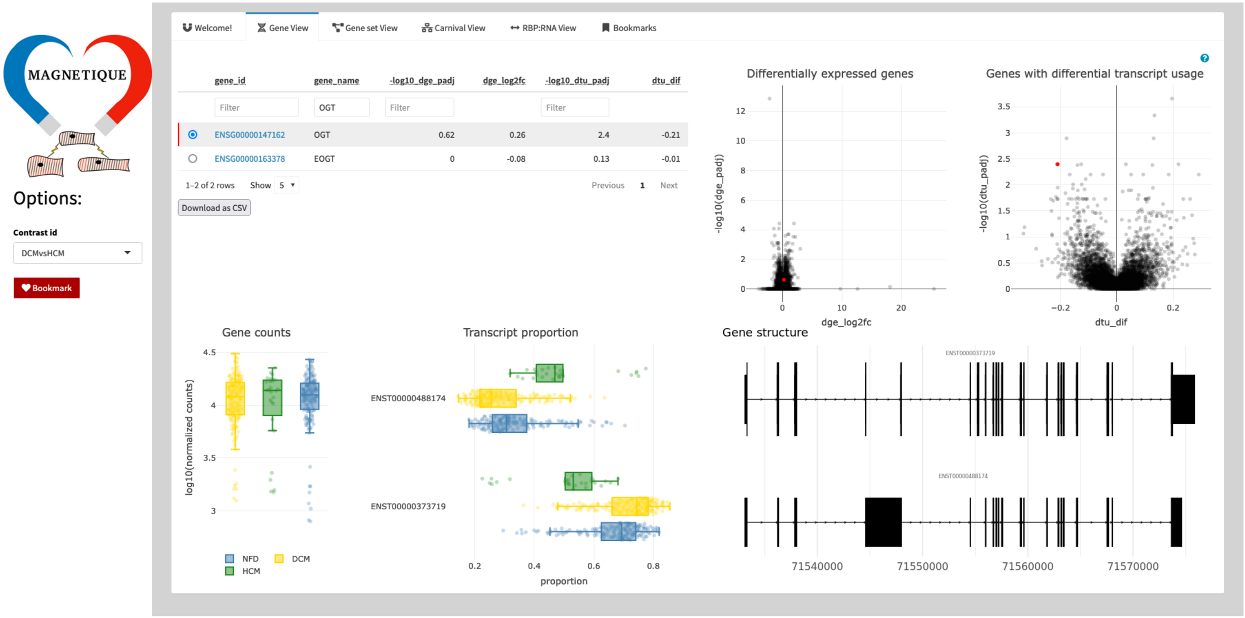

Figure S04: Gene view page. This view presents the summaries for the DGE and DTU analyses. In the top left, the table shows the DGE statistics and summary with the minimum adjusted *p*-value and matching DIF for the DTU analysis. In the top center and right, the volcano plots for DGE and DTU, respectively. At the bottom, the summaries for counts and transcript usage and the transcript structure for transcripts that were tested for DTU are triggered once a row in the table is selected. The option in the sidebar (left) allows users to select the contrast.

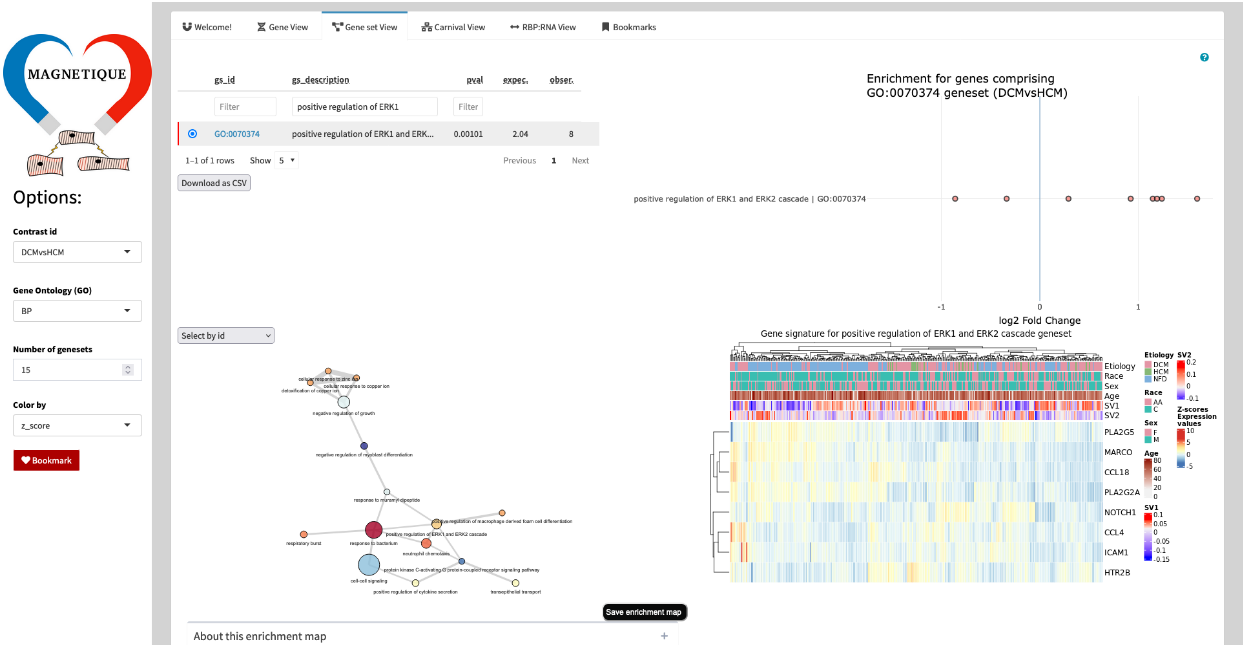

Figure S05: Gene set view. The top-left table lists the terms and statistics for the gene set enrichment analysis with TopGo. The top right plot shows the individual log2 fold changes for genes for multiple terms or a single one if a row in the table is selected. The network on the left

shows the context among different terms; see Figure S09 for details. The heatmap in the bottom right shows gene signature (z-score transformed gene expression) and covariates used for modeling for a given term, once selected from the table. Multiple options can be set on the side panel.

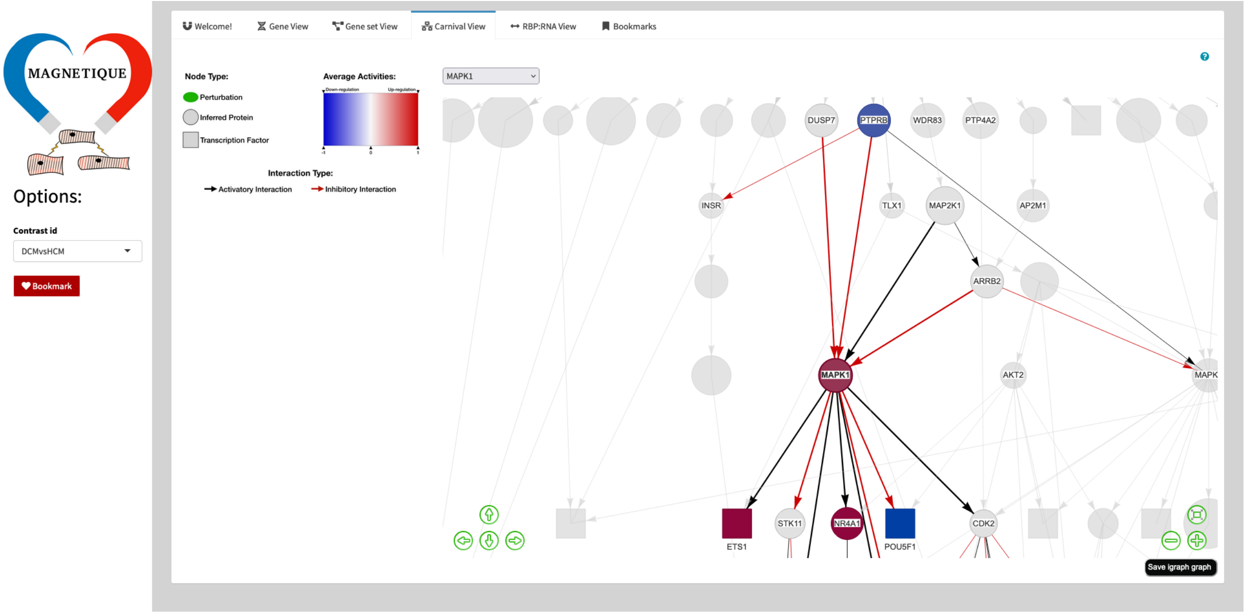

Figure S06: Carnival View. The network shows multiple levels of protein signaling networks and inferred protein activities given the gene expression signature of a contrast (selected in the options). Regulatory interactions represented by the edges (edges) can either activate or inhibit targets. Transcription factors are represented by rectangular nodes.

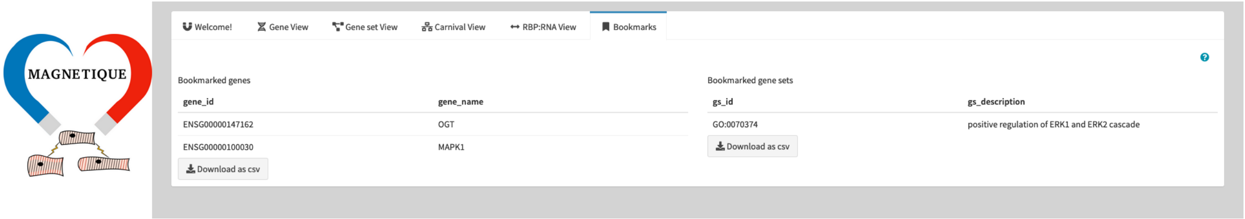

Figure S07: Bookmarks tab. The bookmarks tab shows the bookmarked genes from the Gene View or Carnival View and gene sets from the Gene set View that were selected with the bookmark button. The lists can be downloaded with the "*download as csv"* button.

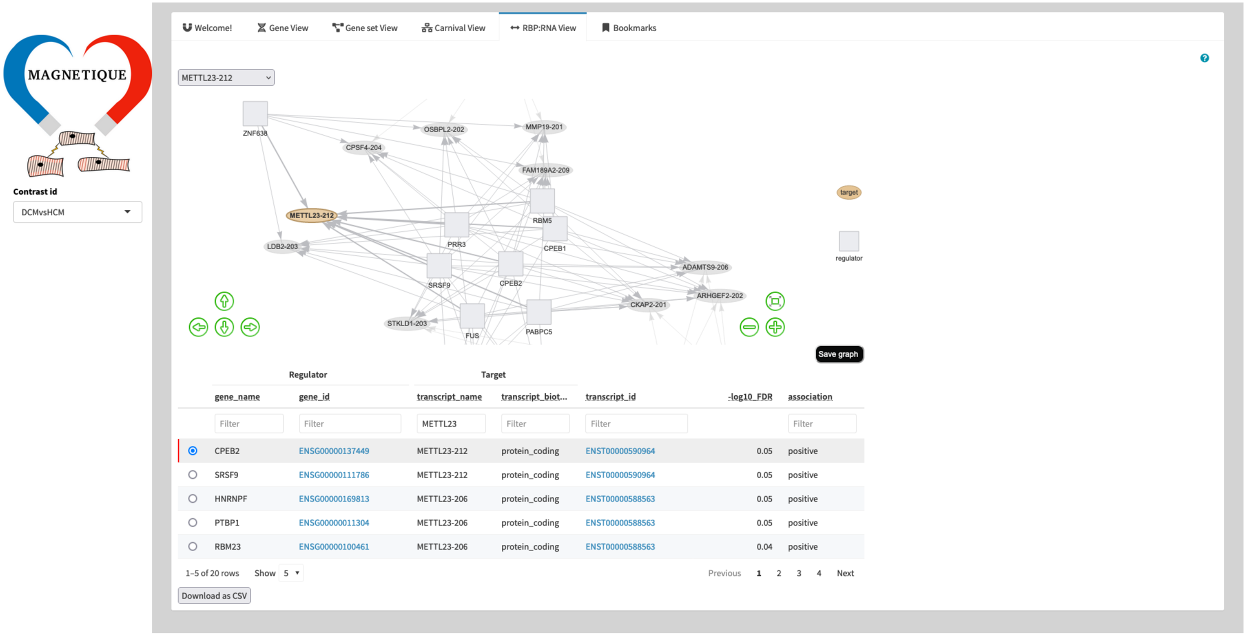

Figure S08 Overview of RBP:RNA View. This view lists the interactions between RBP (regulator) and transcripts isoforms (targets) for genes that host significant DTU events.

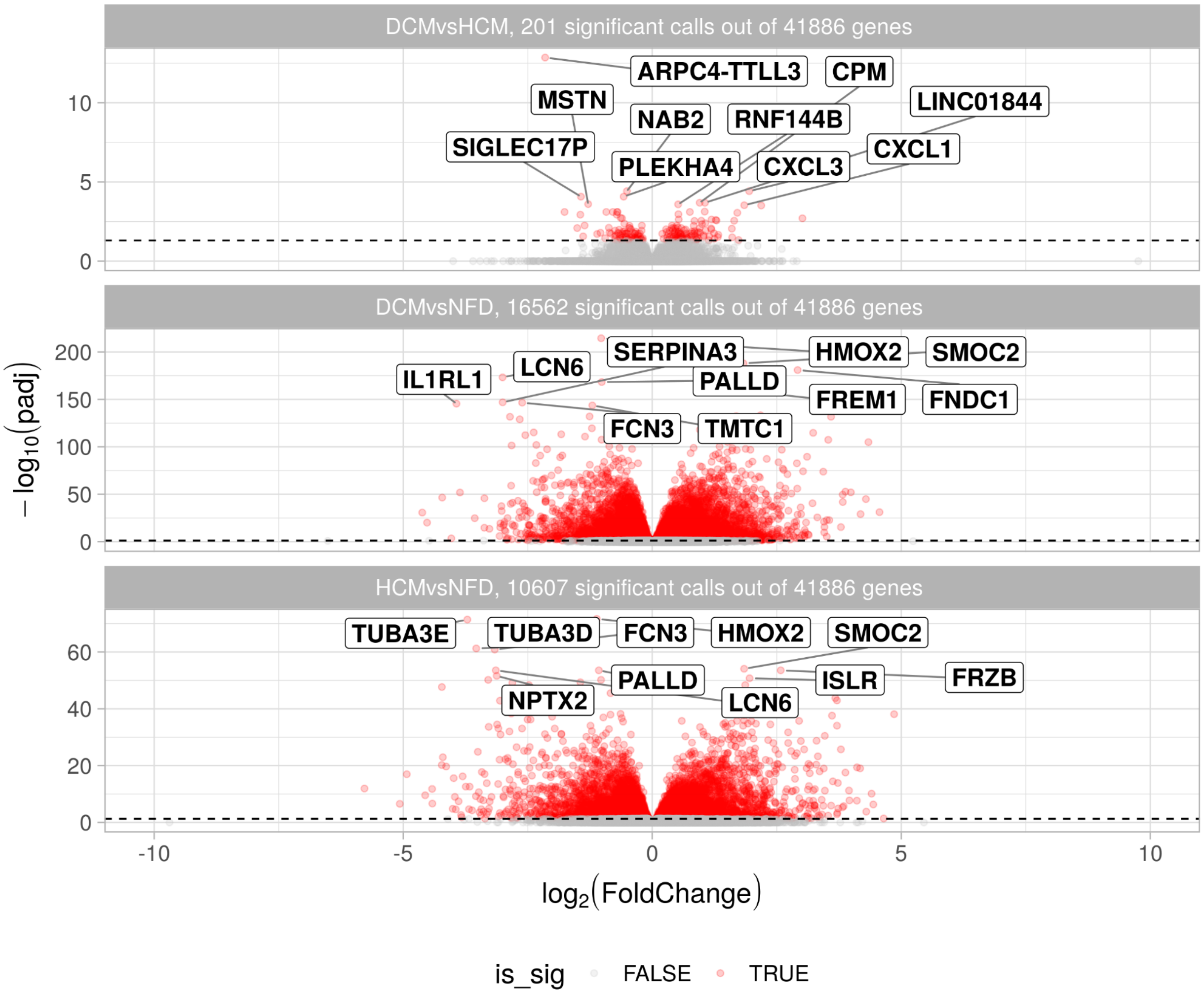

Figure S09: Volcano plot for the DGE analysis. Red circles represent genes called significant (adjusted p-value ≤ 0.05). Top ten genes for each contrast were named with the gene symbol.

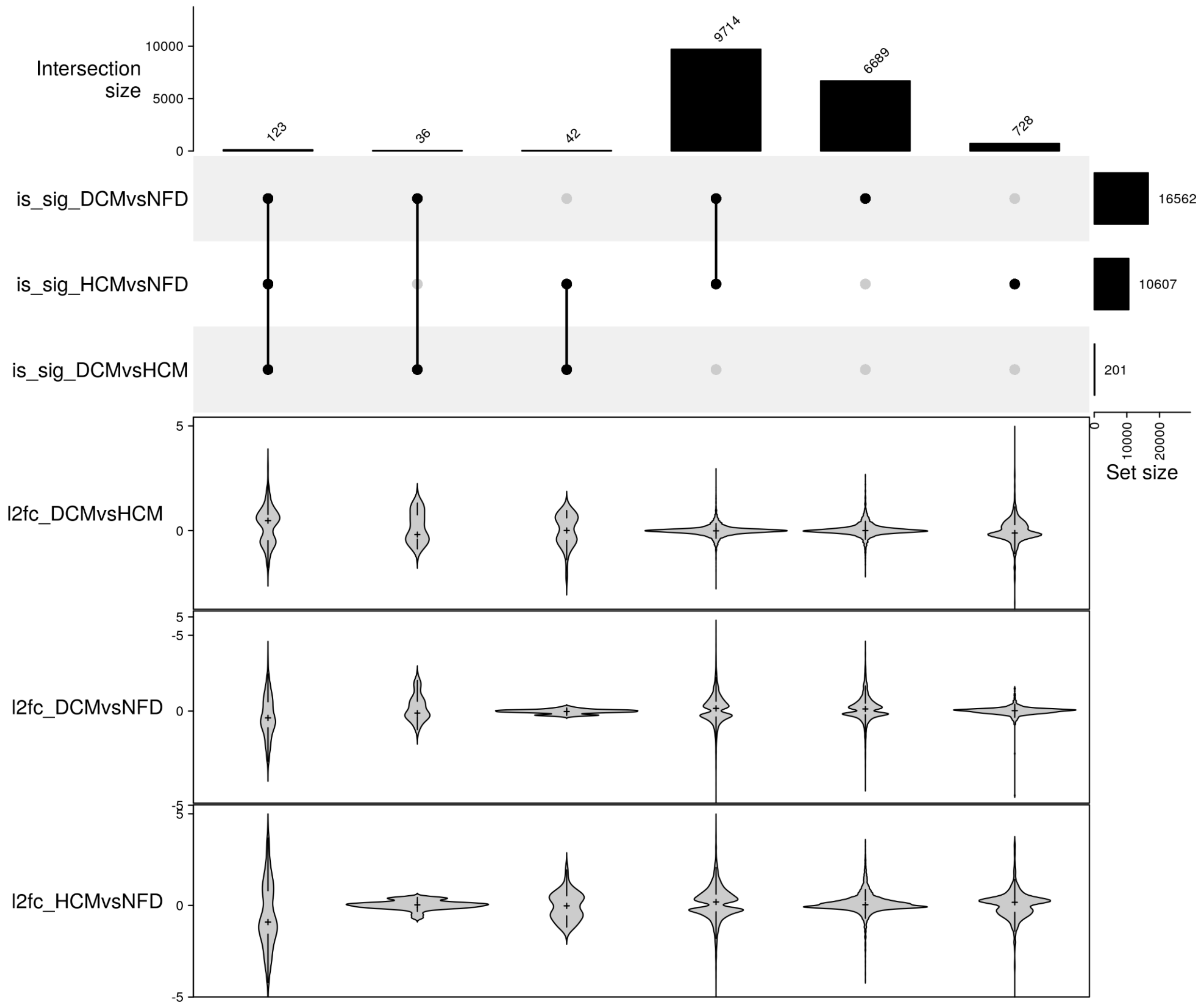

Figure S10: Intersections of differentially expressed genes among contrasts. The UpSet plot comprises three panels: bar plots with the intersection sizes (top), the intersections of genes that were called significant among the three comparisons (middle), and the distributions of the log fold change for each intersection (bottom). Each column represents an intersection among the common genes for each contrast. The bar plot in the middle left shows the total number of genes called significant for each comparison. A total of 123 genes are commonly differentially expressed by the three contrasts. Genes in this combination have a median log2(FoldChange) of 0.48, and so are upregulated.

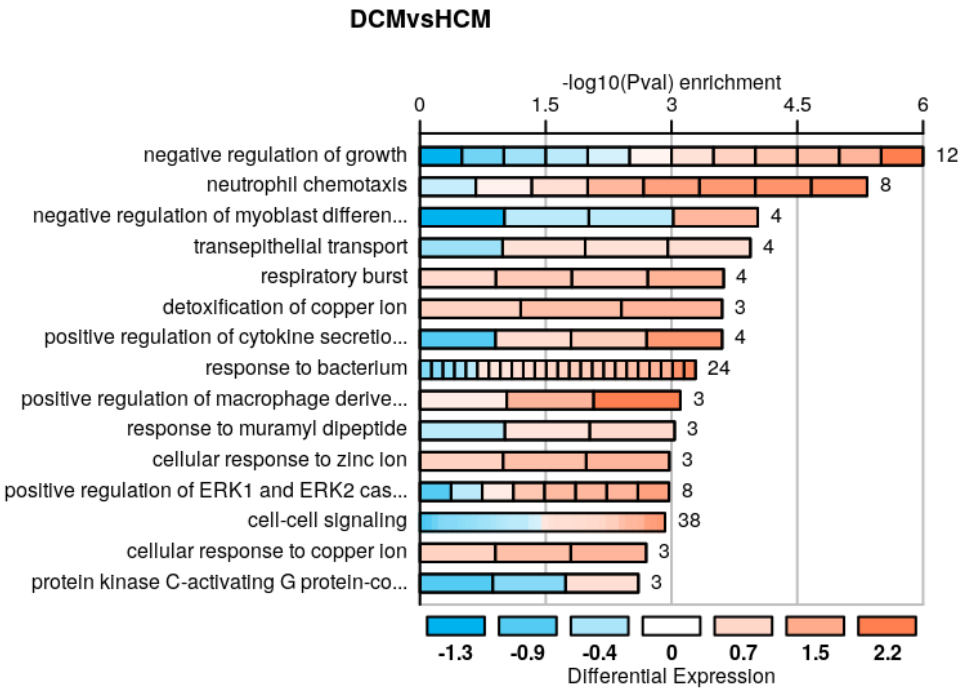

Figure S11: **The top 15 Biological Process terms ranked *p*-value for the DCMvsHCM comparison.** Each cell represents a gene and the cell colors show the effect sizes for the DGE analysis, as annotated in the bottom of the image.

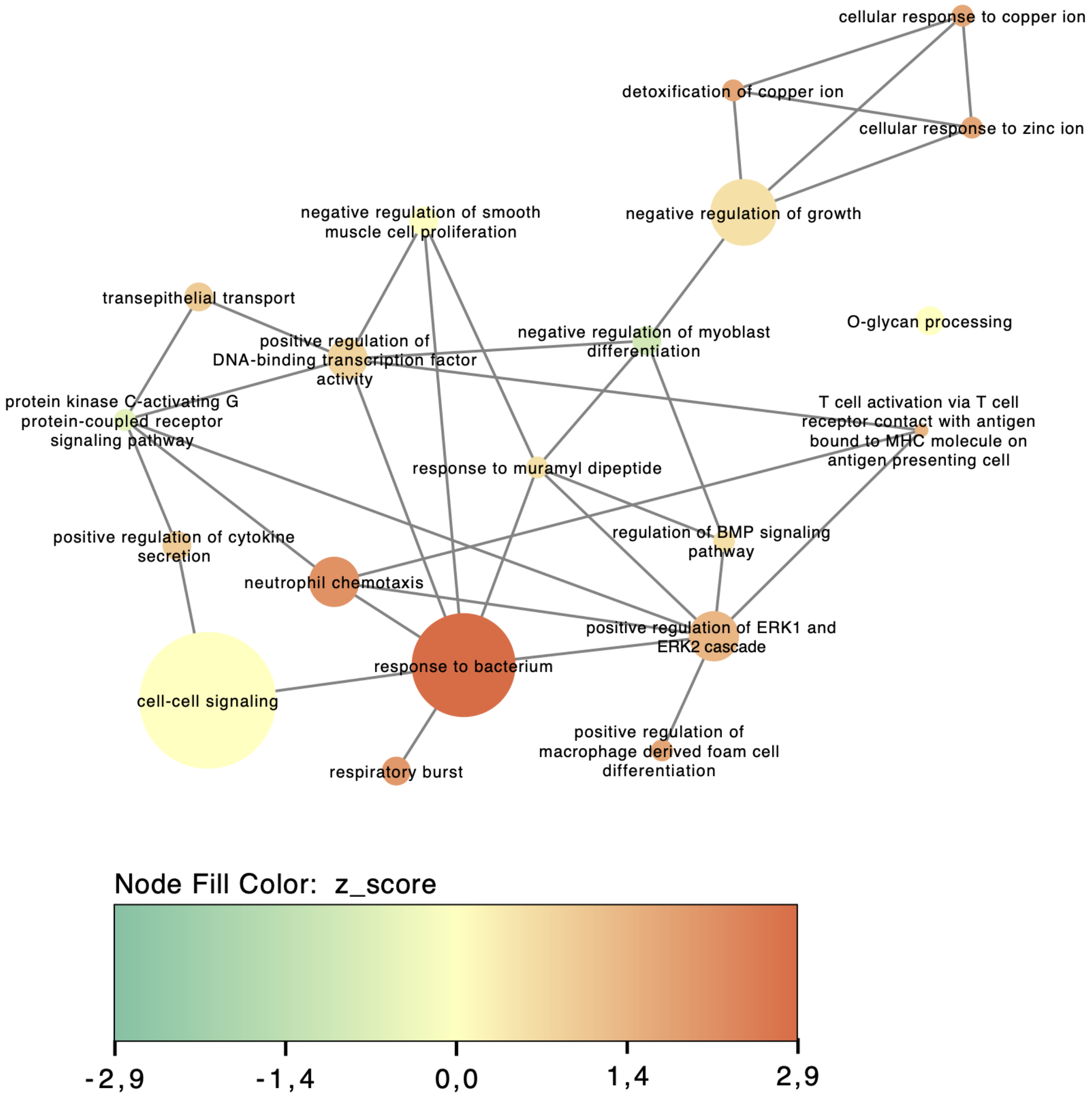

Figure S12: The enrichment map for top biological process terms called significant in the GSEA. Node sizes represent the number of genes per term, and node color represents the average log fold change (z-score transformed) for gene members of each gene set; orange nodes have more genes highly expressed in DCM, while green nodes have more genes highly expressed in HCM. The cell-cell signaling pathway was the term with the highest number of DGE genes. Negative regulation of myoblast differentiation (GO:0045662, more genes highly expressed in HCM) and response to bacterium (GO:0009617, more genes highly expressed in DCM) are terms with more extreme effect sizes.

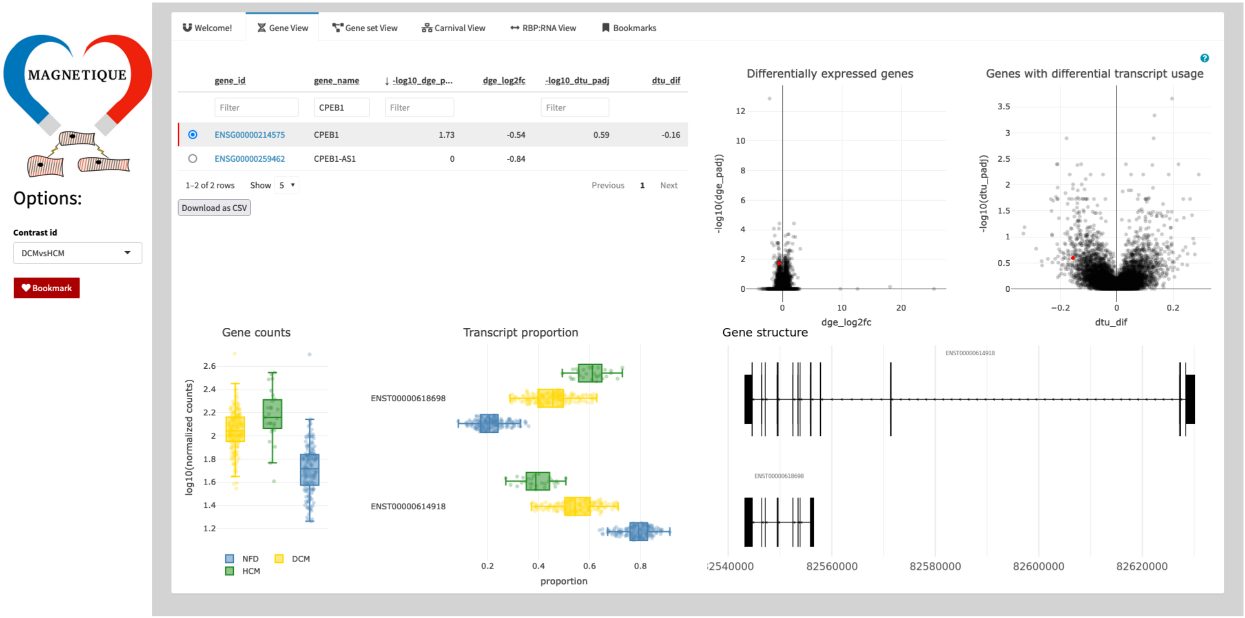

Figure S13 Overview of DGE and DTU for CPEB1. CPEB1, the Cytoplasmic Polyadenylation Element Binding Protein 1. It is differentially expressed for DCMvsHCM comparison, but also undergoes different transcript usage for DCMvsNFD and HCMvsNFD. The two disease condition show the same pattern for isoform usage: the primary use of CPEB1-211 (ENST00000615198) while the longer isoform (CPEB1-209 - ENST00000614918) is preferably used in the NFD.

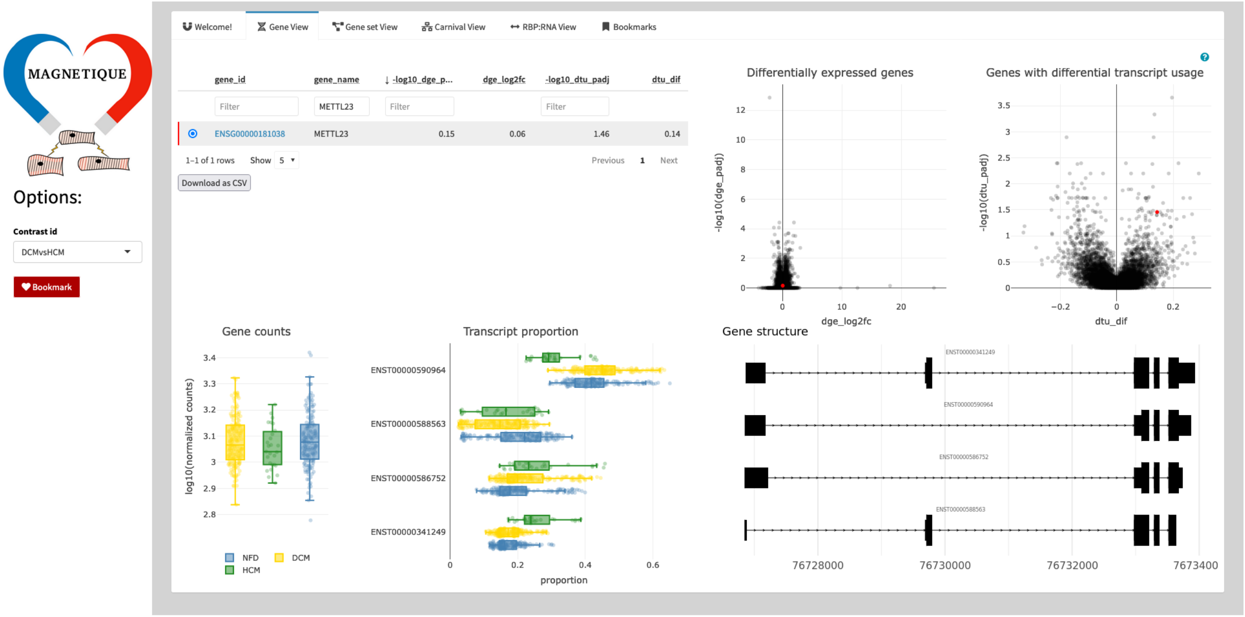

Figure S14 Overview of DGE and DTU for METTL23. Histone-arginine methyltransferase METTL23 is one of the targets that was prioritized by reverse global test analysis and also undergoes DTU.

Gene_id regulator	Gene_name regulator	Transcript_name Target	Transcript_id Target	Transcript_biotype Target	FDR	Association
ENSG00000214575	CPEB1	FAM189A2-209	ENST00000645516	retained_intron	4.7×10^{-2}	+
ENSG00000214575	CPEB1	METTL23-212	ENST00000590964	protein_coding	4.7×10^{-2}	-
ENSG00000214575	CPEB1	CKAP2-201	ENST00000258607	protein_coding	4.7×10^{-2}	-
ENSG00000214575	CPEB1	CPSF4-204	ENST00000436336	protein_coding	4.7×10^{-2}	-
ENSG00000214575	CPEB1	ARHGEF2-202	ENST00000313695	protein_coding	4.9×10^{-2}	+
ENSG00000214575	CPEB1	ADAMTS9-206	ENST00000477180	retained_intron	4.9×10^{-2}	-

Table S04: Targets of CPEB1 with FDR ≤ 0.05 for the reverse global test and adjusted *p*-value < 0.05 for DTU.

	DCMvsHCM	DCMvsNFD	HCMvsNFD
Nodes (proteins)	111	142	92
Edges (interactions)	201	265	164

Table S05: Number of components for the network solutions obtained by CARNIVAL.
